# The Influence of pH on the Specific Adhesion of P Piliated *Escherichia coli*

**DOI:** 10.1371/journal.pone.0038548

**Published:** 2012-06-05

**Authors:** Jeanna E. Klinth, Mickaël Castelain, Bernt Eric Uhlin, Ove Axner

**Affiliations:** 1 Department of Physics, Umeå University, Umeå, Sweden; 2 Department of Molecular Biology, Umeå University, Umeå, Sweden; 3 Umeå Centre for Microbial Research (UCMR), Umeå University, Umeå, Sweden; 4 Laboratory for Molecular Infection Medicine Sweden (MIMS), Umeå University, Umeå, Sweden; Columbia University, United States of America

## Abstract

Adhesion to host tissues is an initiating step in a majority of bacterial infections. In the case of Gram-negative bacteria this adhesion is often mediated by a specific interaction between an adhesin, positioned at the distal end of bacterial pili, and its receptor on the surface of the host tissue. Furthermore, the rod of the pilus, and particularly its biomechanical properties, is believed to be crucial for the ability of bacteria to withstand external forces caused by, for example, (in the case of urinary tract infections) urinary rinsing flows by redistributing the force to several pili. In this work, the adhesion properties of P-piliated *E. coli* and their dependence of pH have been investigated in a broad pH range by both the surface plasmon resonance technique and force measuring optical tweezers. We demonstrate that P piliated bacteria have an adhesion ability throughout the entire physiologically relevant pH range (pH 4.5 – 8). We also show that pH has a higher impact on the binding rate than on the binding stability or the biomechanical properties of pili; the binding rate was found to have a maximum around pH 5 while the binding stability was found to have a broader distribution over pH and be significant over the entire physiologically relevant pH range. Force measurements on a single organelle level show that the biomechanical properties of P pili are not significantly affected by pH.

## Introduction

**Figure 1:**
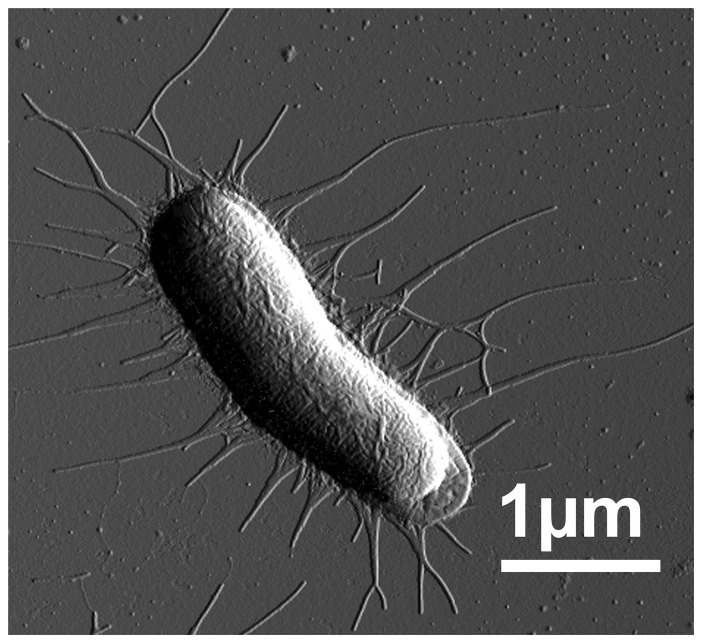
AFM micrographs of HB101/pPAP5 cells expressing P pili.

Bacterial adhesion to host tissue is an early event in the infection process. A wide range of Gram-negative bacteria express adhesive organelles, so-called pili or fimbriae, on their outer surface [1]. In pathogenic strains, pili are an important virulence factor involved in host recognition and attachment [2], [3], in the formation of biofilms [4], and for cell invasion [5]. P pili are prototype helix-like pili expressed by uropathogenic *E. coli* (UPEC) strains (figure 1) that are correlated to pyelonephritis [6]. Adhesion of P piliated bacteria to their human kidney receptors is considered an initial step in the development of pyelonephritis in humans [7]. P pili consist of a rod built by more than thousand copies of PapA subunits in a helical conformation, and a flexible tip with an adhesin (PapG) at its distal end. Throughout the years, this adhesion system has been studied in some detail [2], [7], [8], [9], [10], [11], [12]. For example, it has been shown that in pyelonephretic UPEC strains, the PapG adhesin binds to galabiose [α-D-Gal-(1–4)-β-D-Gal] containing glycolipid receptors on the kidney epithelium [7], [13].

Moreover, with access to state-of-the-art instrumentation – primarily force measuring optical tweezers (FMOT) – it has recently become possible to assess, on a single organelle level, also various properties of the pili rod, in particular its biomechanical properties. It has been found that the response of helix-like pili to external force depends strongly on the quaternary structure of the pili. They show an intricate and extraordinary force-*vs.*-elongation response that consists of a combination of a constant elongation force, originating from a sequential uncoiling of the helix-like structure, and a sigmoidal pseudo-elastic response, caused by a conformational change of the head-to-tail interaction between the subunits of pili that takes place in a randomized order [14], [15], [16], [17], [18]. It has been suggested that this unique non-linear force-*vs*.-elongation response assists piliated bacteria in their adhesion process; that is it plays an important role for bacteria to resist strong shear forces, for example, those from urine flows, by distributing an external force among several individual pili [15], [19]. Hence, although the adhesins play the main role in the initial adhesion and colonization [13], [19], it has been argued that the rod of the pilus is of significant importance for the ability of bacteria to withstand forces caused by urinary rinsing flows.

Because of its important role in bacterial virulence, the entire helix-like pilus has become coveted targets for novel antibacterial agents. Several methods for impairing the adhesion ability of piliated bacteria have been attempted. For example, it has been shown that derivates of galabiose (galabiocides) can act as potent competitive inhibitors of PapG [20], [21], [22], [23]. In addition to such substances, compounds affecting the pilus assembling process (pilicides) have been identified. Pilicides, such as rationally designed bicyclic 2-pyridones, block the subunit binding site in the chaperone and prevent pili formation [24], [25], [26], [27]. An alternative anti-adhesion strategy is interference of polysaccaccharides with quorum sensing [28], [29].

It can be assumed that the surrounding environment has an impact on bacterial survival. For example, it has been demonstrated that urine is an excellent bacterial culture medium [30], [31]. On the other hand, it has been demonstrated that urine also has a bactericidal effect on Gram-negative bacteria. Antibacterial activity has been correlated with, for example, osmolarity, concentration of urea, ammonium and acid mucopolysaccharide (AMPS), and pH. For example, it has been noted that urine at pH values of 5.0 and below is often inhibitory for growth of microorganisms [31], [32], [33]. Several components have therefore been tested for their ability to acidify urine [34], [35]. In addition, a number of studies, most of them performed several decades ago, were therefore aimed to enhance the activity of antibiotics by an alteration of the urine pH. Although it was found that the tested antibiotics indeed have a pH dependence, it was found that they often have their optimal activity under slightly alkaline conditions [33]. Since this does not correlate to the observed pH dependence of the growth of microorganisms, and since the majority of antibacterial drugs act intracellularly, the pH of urine can be assumed to be of less importance for such drugs.

On the other hand, as the adhesion organelles are expressed on the outer surface of bacteria, it can be surmised that the influence of the environment is relevant for the functioning of these organelles. For example, it has been shown that pH has an effect on *Helicobacter pylori* binding to human gastric mucins [36]. The pH of the gastric mucus layer varies from acidic in the lumen to neutral at the cell surface. Similarly, since the pH of human urine varies in a broad range (variations of pH in human urine and gender differences in urinary pH have been reported [31], [37]), it is of importance to clarify to which extent pH can affect the adhesion ability of UPEC bacteria. We therefore decided to address this question using methodology that would allow studies at the levels of single bacterial cells and of single adhesion organelles.

In this study, we have investigated various adhesion properties of P-piliated *E. coli* in a broad pH range in order to assess their dependence on pH. The specific adhesion of P-piliated *E. coli* to a galabiose-coated surface was assessed in real time using the surface plasmon resonance (SPR) technique while the biomechanical properties of P pili were monitored on a single organelle level using force measuring optical tweezers (FMOT). We show that P piliated bacteria have an adhesion ability throughout the entire physiologically relevant pH range (pH 4.5 – 8). It is also demonstrated that whereas the specific adhesion rate of P-pili have a pronounced pH dependence, with a maximum at around pH 5, the binding stability and the uncoiling force of the quaternary structure of the pilus rod are only marginally affected by alterations in the pH.

## Results

### Surface Plasmon Resonance (SPR) assays

**Figure 2:**
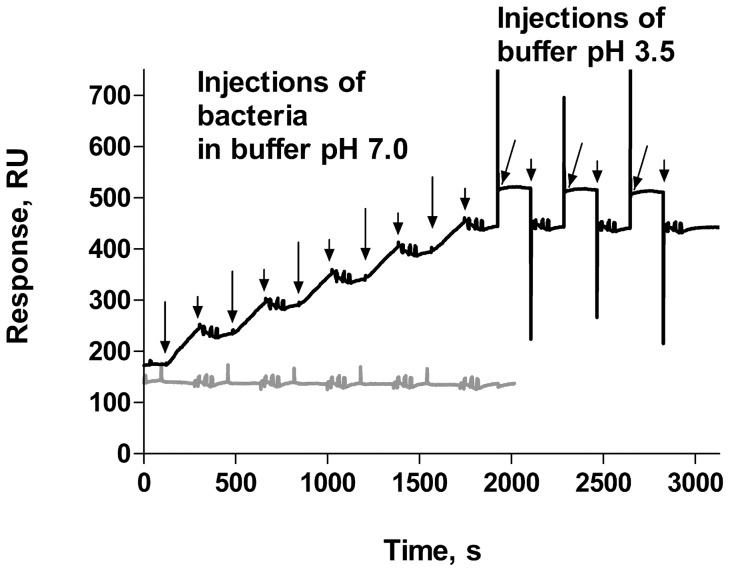
Binding of *E. coli* expressing P pili and type 1 pili in SPR assay. Each type of bacteria (P pili-black line and type 1 pili-gray line) was injected into the flow chambers five times and washed with buffer at pH 7.0 between each injection. P-piliated bacteria were thereafter exposed to three injections of low pH buffer (3.5). The sensogram shows the difference in response between the galabiose-coated and the BSA-coated cells. Large arrows indicate start of injection and short arrows indicate end of injections. The alternating levels during injections of buffer with pH 3.5 originate from different index of refractions for the two buffer solutions (pH 3.5 and 7, respectively).

The interaction between the PapG adhesin on the tip of P pili and its globoside receptor on the uroepithelium of the human kidney is known to be highly specific [7]. In our study, we used a galabiose-bovine serum albumin (BSA) conjugate to investigate the influence of pH on the specific bacterial adhesion in real time using label-free interaction analysis in an SPR assay. BSA-coated surfaces were used as references. The signals from the reference surfaces were considered as background and were subtracted from those from galabiose-coated surfaces before the binding rates and binding specificities were evaluated. The assay conditions were chosen according to the work by Salminen *et* al. [21]. The binding ability (response) of two types of UPEC bacteria, expressing different types of pili (P pili and type 1), is shown in figure 2. As shown by the first part of the black curve (up to 1800 s), we found that there was a gradual increase of the binding of *E. coli* expressing P pili to a galabiose-coated surface when bacteria was injected five repetitive times at pH 7.0, whereas no significant response was observed after injection of *E. coli* expressing type 1 pili (grey curve). In order to investigate the binding stability of the attached bacteria the P pili samples were thereafter exposed to a low pH. As illustrated by the last part of the black curve, the binding was stable – the signal decreased only marginally after three injections of buffer with low pH (pH of 3.5, by ∼ 1%).

**Figure 3:**
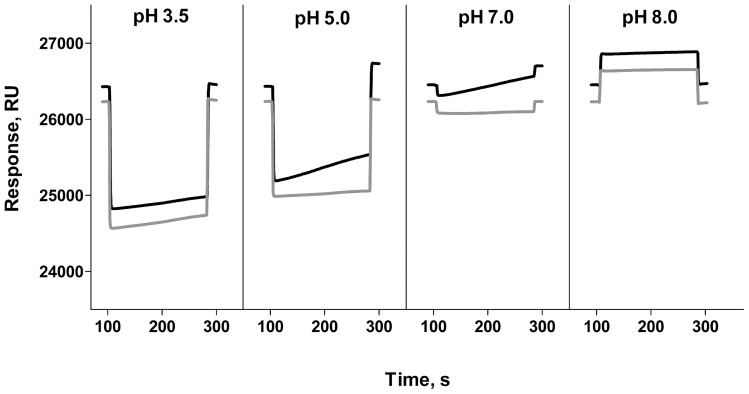
Binding of P pili in SPR assay at four different pH. Black lines show pili binding to galabiose-coated surface and gray line – to BSA-coated surface.

At the prevalent conditions both whole bacteria and isolated pili showed a linearly increasing binding to surfaces with time. Figure 3 demonstrates typical binding responses of P pili to a galabiose-coated and a reference surface (black and gray line, respectively) at four different pH values. Since the responses were found to be linear with time, their slopes could be used for assessment of binding rates.

**Figure 4:**
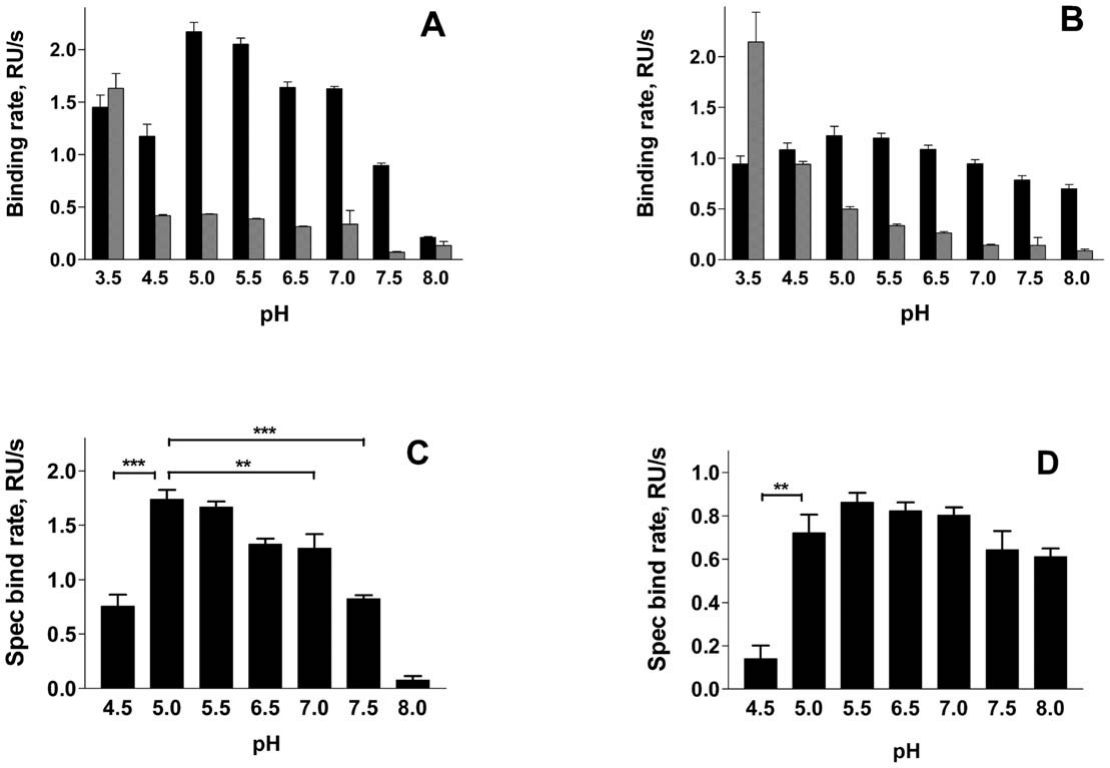
Calculated binding rates of pili and bacteria at different pH. Panels A and B show the binding to galabiose-BSA-coated surface (black bars) and BSA-coated surface (gray bars) for pili and bacteria respectively. Panels C and D show the specific binding rates of pili and bacteria respectively, calculated as difference between binding to galabiose-BSA-coated surfaces and BSA-coated surfaces obtained in SPR experiments.

Binding rate experiments were performed on isolated P pili as well as whole bacteria at several different pH values, ranging from 3.5 to 8.0. The assessed binding rates for pili and for bacteria to galabiose-coated and BSA-coated surfaces (black and gray bars, respectively) are shown in the figures 4A and B respectively. These panels illustrate that the binding rates for both pili and bacteria to galabiose-coated surfaces are, for the entire physiologically relevant range of pH (4.5 – 8.0), consistently higher than those to the reference surfaces. However, at pH 3.5 there were more binding to BSA-coated surfaces than to galabiose-BSA, which is interpreted as excess nonspecific binding. The SPR results at this pH level were therefore in the following discarded.

The specific binding rate of isolated pili, calculated as the difference between the binding rates at the galabiose-coated and the reference surfaces, is shown in figure 4C. It was found that this rate was higher at pH 5.0–5.5 as compared to at other pH values: the binding rate at pH 7.0 was 25% lower than that at pH 5.0 (p<0.01). The corresponding numbers for pH 4.5 and 7.5 were 50% (p<0.001) and for pH 8.0, 5% (p<0.001). This is in good agreement with previous studies showing that purified adhesin (PapG) causes hemagglutination and agglutination of galabiose-coated latex beads with an optimum at pH 5.0 [38].

As is shown in figure 4D, for whole bacteria, only a weak dependence of the specific binding rate on pH was observed for a large part of the pH range investigated (pH 5 – 8). The only significant dependence on pH was found at low pH values; at pH 4.5 the rate was found to be only 25% of that observed at higher pH values (p<0.01).

**Figure 5:**
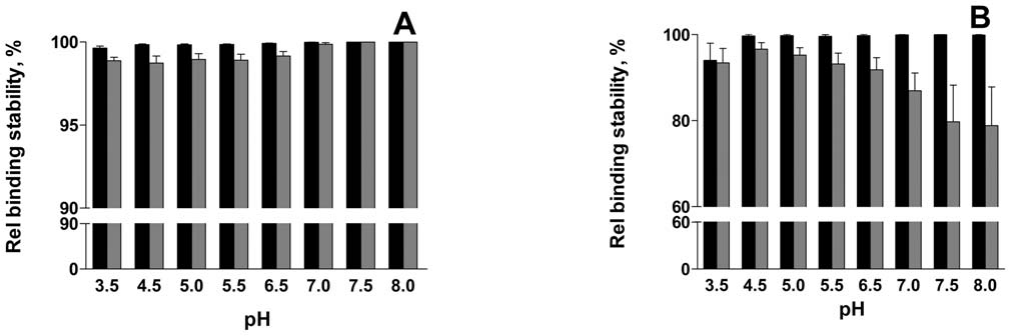
Relative binding stability of pili (A) and bacteria (B). Black bars represent the remaining amount of pili and bacteria on galabiose-coated surfaces and gray bars on BSA-coated surfaces at different pH measured after a wash of the surfaces with a buffer at pH 7.0. 100% corresponds to the amount of bacteria on the surface before the wash.

As mentioned above, and as shown in figure 2, bacteria bound to galabiose-coated surfaces at pH 7.0 remained attached after subsequent exposure to a low pH (3.5). The binding stability of bacteria attached to surfaces also at other pH values was then investigated. The relative binding stability of both free pili and bacteria was determined as the differences of the SPR responses at two time points after the unbound bacteria had been washed with buffer at pH 7.0 at four minutes intervals. The relative binding stability was defined as: (bound – detached)/bound × 100%. As shown in figure 5, it was found that the binding stability of both isolated pili and bacteria to galabiose-coated surfaces was high; it was close to 99% for isolated pili in the entire pH range investigated (pH 3.5 – 8.0, figure 5A, black bars) whereas for bacteria it was likewise high in most of the pH range investigated (pH 4.5 – 8.0, figure 5B, black bars) although slightly smaller (∼95%) for the lowest pH value (3.5). Bacterial binding to BSA surfaces (grey bars) was less stable than to galabiose-coated surfaces.

**Figure 6:**
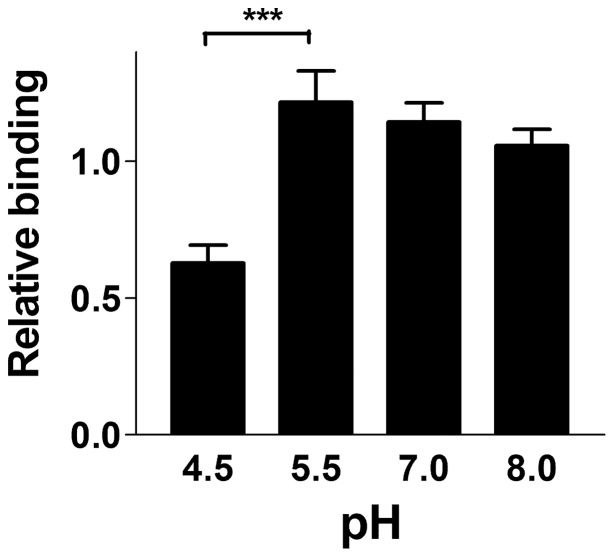
Relative binding of P piliated *E. coli* in artificial urine medium when compared to binding in buffer at pH 7.0.

Finally, the effect of pH on bacterial binding in artificial urine medium (AUM) was studied at four different pH values: 4.5, 5.5, 7.0 and 8.0. AUM is a complex medium that provides conditions similar to that found in human urine [39]. The total binding of bacteria to galabiose-coated surfaces in AUM was, for each pH value, compared to the total binding in buffer at pH 7.0. Two different strains of *E. coli* were used. Using two-ways analysis of variance (ANNOVA), no significant difference between the binding of these two strains was found. The results of the binding of HB101/pPAP5 are shown in figure 6. Despite the complexity of AUM, a binding pattern similar to experiments in buffer was found (figure 4D).

### FMOT measurements

**Figure 7:**
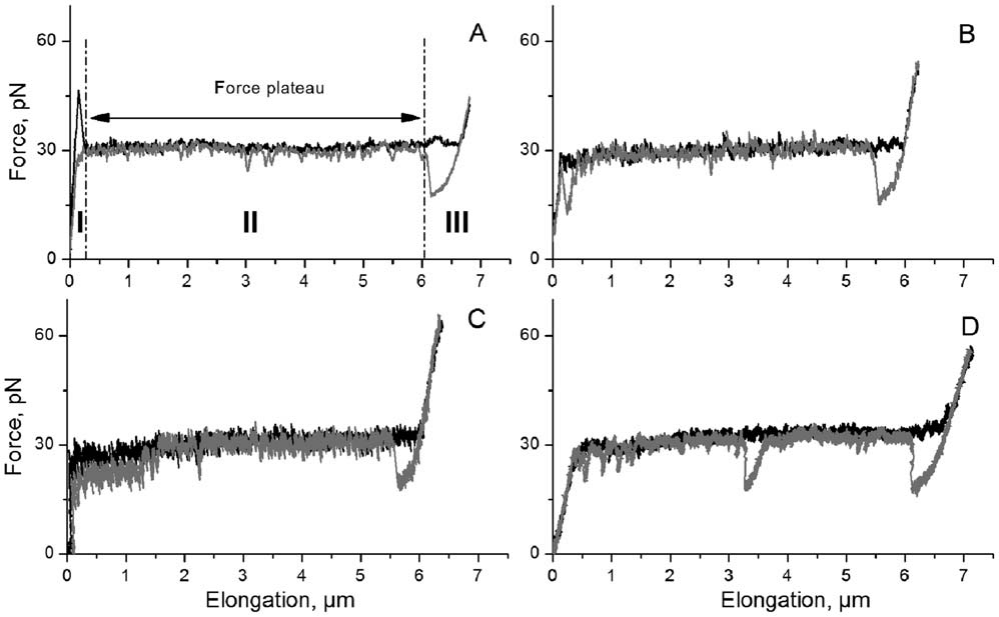
Force-*vs.*-elongation response of a single P pilus at different pH. Panels A-D show the elongation at pH 7.4, pH 5.0, pH 3.0 and pH 8.0 respectively. The plateaus of the black curves illustrate uncoiling forces of a single pilus, and the gray curve the recoiling forces. The three regions of force-*vs*.-elongation response are marked with Roman numerals (I–III), and the characteristic force plateau (region II) is indicated with a double arrow in (A).

**Figure 8:**
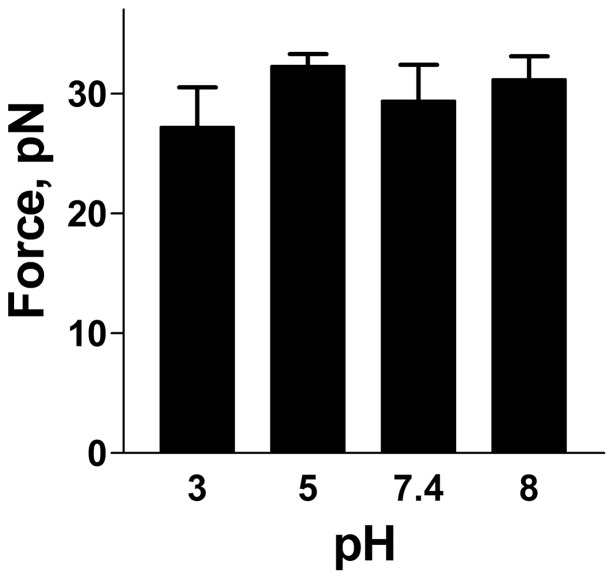
Uncoiling forces at different pH in FMOT experiments.

Since it has been argued that the ability of attached bacteria to withstand forces caused by urine flows does not only depend on properties of the adhesin-receptor bond, but also on the biomechanical properties of the pilus rod [15], [18], [19], [40] the influence of pH on the uncoiling forces of the pilus rod was examined using FMOT. As shown in figures 7 A–D, which display the force-*vs*.-elongation forces of a single P pilus elongated at pH values 8.0, 7.4, 5.0 and 3.0, the response is composed of three regions (I–III, figure 7A) [16]. Region II shows a constant force response (a force plateau) that is specific for a certain type of pili [17]. This force plateau originates from a sequential breaking of the layer-to-layer interactions of the quaternary structure of the pilus rod in an uncoiling process. Force-*vs*.-elongation curves of P pili at different pH values are shown in figure 7A–D. The mean values of the uncoiling force of P pili at the four pH values, which are summarized in figure 8, were found to be 31±1.9 (n = 26), 29±3.0 (n = 29), 32±1.0 (n = 35) and 27±3.3 (n = 15) pN respectively (where the variation stands for the standard deviation, SD). Although an analysis of the variance (by one-way ANNOVA) shows that the uncoiling force at pH 5 is slightly higher as compared to other investigated pH values, the data mainly indicate that the pili maintain their biomechanical uncoiling-recoiling properties throughout the entire pH range investigated (notably including the lowest pH value, 3.0).

## Discussion

Due to their ability to increase the acidity of urine, cranberry juice and ascorbic acid have been suggested as prophylaxis against urinary tract infection. These assumptions have also been confirmed by clinical studies [41], [42], [43]. In this work we demonstrate the effect of pH on the adhesion properties of isolated P pili as well as P pili expressing *E. coli*. The specific binding rate and the binding stability of P pili, determined by the adhesin in the distal end of their pili, were studied using the SPR technique where the specific receptors, galabiose, were immobilized to the surface. Studies were made on both isolated pili and piliated bacteria. Moreover, we assessed the biomechanical properties of the pili, since it has been conjectured that they play an important role in the adhesion process of bacteria. For these studies we used the FMOT technique to address single pili on individual bacterial cells (because of its previously demonstrated applicability to scrutinize the biomechanical properties of helix-like pili [14], [15], [16], [17], [18]) and to mimic shear forces in vitro.

In this work we demonstrate that P piliated bacteria have an adhesion ability throughout the entire physiologically relevant pH range (pH 4.5 – 8). At pH values below 4.5 the specific binding ability dramatically decreases, presumably because of an overall effect on the 3D structure of the protein by a lowering of the pH value rather than a change of the binding pocket of PapG. A number of amino acids such as Arg, Lys, Glu, Asp, Trp and Tyr have previously been found to be important for the adhesin-receptor interaction [7]. Those amino acids interact with the galabiose by hydrogen and hydrophobic interactions, which are independent of pH variations. Furthermore, the structure of galabiose is uncharged, whereby no salt bridges to the protein are created. Thus, most likely the salt bridges of PapG located elsewhere (not in the binding pocket) are affected by a lowering of the pH, causing a conformational change of the entire 3D protein structure so the adhesin loses its ability to bind specifically to its receptor.

The specific binding ability as well as the binding rate of P pili has a maximum at pH values of 5.0 and 5.5 (figure 4A and C). At pH 8.0 the specific binding rate of individual pili was found to be significantly lower, only 10% of that at pH 5. Despite this fact, it was found that whole bacteria have almost the same binding ability and rate throughout the entire physiologically relevant region, i.e. from pH 5.0 to 8.0 (figure 4B and D). We interpret the difference between these two responses to an avidity effect. Since the bacteria express a multitude of pili, and it is sufficient that solely one of these pili adhere to give rise to a response in the SPR technique, it is plausible that conditions that provide a variety of binding rates of isolated pili give rise to similar adhesion abilities (and binding rates) of whole bacteria.

Moreover, at the lowest pH value (3.5), the unspecific binding rate of whole bacteria was found to be considerable. This is assumed to originate from an increased electrostatic interaction between the bacteria and BSA on the surface. This effect does not presumably take place in vivo conditions where galabiose receptors have a more complex environment.

The specific binding rate of both pili and bacteria was found to be extraordinarily stable at all the investigated pH values (figure 5). The stability of specifically bound *E. coli* expressing P pili was high (99%) for a large range of pH (from 4.5 to 8). In fact, it was even found that although whole bacteria do not bind to galabiose-coated surfaces at pH 3.5, bacteria that originally bound at higher pH levels remained attached after repeated exposure to buffer with pH 3.5 (figure 2). This indicates that the SPR measurements are performed under conditions for which the rate of bacterial detachment is low, thus presumably also under conditions for which no uncoiling takes place. The high stability of specifically bound P pili is in good agreement with a previously reported long dissociation time of bacteria by Pourshafie *et* al. [44], who reported on slow dissociation kinetics between glutaraldehyde-fixed bacteria and their receptor (dissociation time ∼6 h). Since live bacteria were used in the present study, this implies that the time for analysis was limited by the generation time of bacteria rather than their specific adhesion time. For comparison, the unspecific binding of whole bacteria (figure 5B, gray bars) decreases with increased pH. Unfortunately, most of the study of the binding stability had to be performed at a neutral pH value because of instrumental limitations – only short time exposures to low pH could be allowed. However, repeated pulses of buffer with a pH value of 3.5 did not significantly affect the bound bacteria.

As was alluded to above, it has previously been shown that not only the adhesion but also the rod of the pilus plays an important role in the bacteriás adhesion process (the uncoiling capacity of the helix-like structure of pilus rod is considered crucial for the ability to withstand external forces from urinary rinsing flows). This work shows that pH has a small or no effect on the biomechanical properties of P pili. The pilus rod remains functional at a pH value of 3.0, even though the uncoiling force is slightly lower there (figure 8). This indicates that the pH does not have any appreciable effects on the biomechanical properties of P pili.

It is important to consider the fact that pH values below 5.0 are infrequent in human urine and that acidification of the human urine to such an extent is known to be a true challenge. A study of Waters *et* al. showed that the mean pH value of the urine from males is slightly lower than that of the urine from females, 5.6 and 5.7 respectively, both well above 5.0 [37]. Furthermore, the mean pH value of urine obtained from pregnant women was significantly higher (6.3) than that of the non pregnant and the urine pH was raised in each trimester of pregnancy [31]. It has also been found that urine from pregnant females supports multiplication of *E. coli* better than urine from non pregnant females, and urine from females tends to support multiplication of *E. coli* better than urine from males. Those differences were related to the differences in pH [31]. However, no significant effects of age on pH was found [37].

In order to acidify the urine using drugs, the most efficient treatment was found to be hydrochloric acid. Using this substance, a lowering of the pH value down to 4.6 has been observed [34], while other tested substances (e.g. ascorbic acid, ammonium chloride, Azidole-Pensin) lowered the pH only marginally [35]. It is worth to notice though that hippuric acid, which is an antibacterial active substance in cranberries, inhibits bacterial growth at pH values of 5.2 or less [45]. However, large amounts of cranberries (350 g) need to be digested for a decrease of the pH value in urine from 6.3 to 5.3 [46]. All this suggests that it is non-trivial to lower the pH of urine below pH 5 even by treatments; the pH value of human urine is thus normally above pH 5. Moreover, the preventive effect of cranberries is still controversial. Some clinical studies disagree regarding its preventive effect [47], [48].

In conclusion, this study shows that P piliated *E. coli* have an adhesion ability throughout the entire physiological pH range. Although the binding rate of isolated P pili has a maximum at pH values 5.0–5–5, it was found that the binding rate of whole bacteria shows a less pronounced pH dependence. The specific binding stability of both P pili and whole bacteria were extraordinarily high also after exposure to an exceptionally low pH value (3.5). Moreover, it was found that the biomechanical properties of P pili are not significantly affected by pH. This demonstrates first of all a wide bacterial adaptability to environmental variations. Secondly, since neither the adhesin-receptor interaction nor the biomechanical properties of P pili are significantly influenced by pH, it also demonstrates that an alteration of the pH does not seem to be a viable means for controlling of bacterial infections caused by P pili expressing bacteria; instead, other means must be explored. One such approach is inhibitors targeting either the adhesin receptors, the biogenesis of pili, or recently described coilicides that impair the biomechanical compliance of P pili [49].

## Materials and Methods

### Bacterial culturing and imaging

The *E. coli* strain expressing P pili was HB101/pPAP5, which is a clone carrying the wild type *pap* gene cluster from UPEC strain J96 [50]. The strain HB101/pPKL4 carries the type 1 pili gene cluster [51]. Bacteria were cultured on trypticase soy agar (TSA, Becton, Dickinson and Company, NJ USA), supplemented with 50 µg/ml carbenicillin (Duchefa Biochemie, Limhamn, Sweden) at 37°C overnight. The expression of pili was confirmed by atomic force microscopy (AFM) imaging as described in [52] and shown in figure 1. Bacteria were re-suspended in phosphate-citrate buffer in a pH range 3.0 – 8.0 (McIlvaine's buffer system [53]) just before use in force measurements.

### Isolation of pili

P pili were purified according to a modified procedure of Gong and Makowski [9]. Briefly, *E. coli* HB101/pPAP5, grown overnight on TSA at 37°C, were harvested and re-suspended in 20 ml cold 5 mM Tris-HCl solution (Sigma-Aldrich, Schnelldorf, Germany; pH 8.0). The pili were detached by shearing with a homogenizer, and then cells and debris were centrifuged. The pili were precipitated overnight with ammonium sulfate (55%) and collected by centrifugation. The pili were washed three times with 0.5 mM Tris-HCl (pH 7.5), re-suspended in the same buffer, and dialyzed overnight. The pili were centrifuged again and filtered through a 0.2-*μ*m low-protein binding filter (MILLEX-GV, Millipore, Billerica, MA).

### Galabiose–BSA conjugate

Galabiose-BSA conjugate was obtained from Lundonia Biotech AB (Lund, Sweden, lot 759UNO1003151). Each galabiose-BSA conjugate contains on average 30 galabiose moieties per BSA.

### SPR assay

Binding of whole cell *E. coli* HB101/pPAP5 and purified P pili was studied using a Biacore X100 (GE Helthcare Bio-Science AB, Uppsala, Sweden). Galabiose-BSA conjugate in coating buffer (10 mM sodium acetate, pH 4.0) was immobilized on a sensor chip CM3 (GE Healthcare Bio-Science AB, Uppsala, Sweden) using the amino coupling kit according to instructions provided by manufacturer (GE Healthcare Bio-Science AB, Uppsala, Sweden) to a final level of 1170 resonance units (RU). As a reference, the second cell of the chip was coated by BSA (Sigma-Aldrich, Schnelldorf, Germany) in coating buffer (10 mM sodium acetate, pH 5.0) to the level of 1000 RU. The binding ability of bacteria and pili in citrate-phosphate buffer (pH 3.5 to 8.0) was studied at 25°C. The buffers were mixed using 0.1 M citric acid and 0.2 M disodium phosphate (both Sigma-Aldrich, Stockholm, Sweden). Bacterial suspension (100 µl, OD_600_ of 0.8) was injected to the flow cells at the flow rate of 30 µl/min. After one minute of dissociation, the chip was flushed with 100 µl citrate-phosphate buffer (pH 3.5) and finally regenerated using 4 M MgCl_2_ (Sigma-Aldrich, Schnelldorf, Germany) [21]. The *E. coli* strain HB101/pPKL4, expressing type 1 pili, cultured using the same conditions as for HB101/pPAP5, were used as a negative control. The data of bacterial binding in buffer were collected from 11 series of measurements performed on four different days using fresh bacteria each day.

Isolated pili were diluted using citrate-phosphate buffer to a concentration which gave a binding to the sensor chip about 250 RU at pH 7.0. The same dilution factor was used for all samples. The binding assay was performed in the same way as the assay with whole bacteria. The experiments were performed in 14 series of measurements performed on three different days.

Two strains of *E. coli* (HB101/pPAP5 and HB101/pHMG93) were used for binding experiments in artificial urine medium [39]. The data of bacterial binding in buffer were collected from 14 series of measurements performed on four different days using fresh bacteria each day.

The running buffer in all experiments was citrate-phosphate buffer (pH 7.0) with addition of 0.005 % polysorbate 20 (GE Healthcare Bio-Science AB, Uppsala, Sweden). Binding levels in each experiment were determined as a difference between the measuring cell (galabiose-BSA) and the reference cell (BSA).

### FMOT measurements

Samples were prepared using polymeric microspheres (9.7 µm, Duke Scientific Corp., Palo Alto, CA) that had been immobilized by heating at 60°C for 1 hour on microscope cover glasses (VWR) and functionalized with 0.01% poly-L-lysine (Sigma-Aldrich, Stockholm, Sweden). On top of these spheres samples containing bacteria and 3.0 µm polymeric microspheres (Duke Scientific Corp., Palo Alto, CA) were applied in total volume of 10 µl and enclosed by a string of high vacuum grease (Dow Corning Corporation, Midland, MI) and a second cover glass (VWR, Stockholm, Sweden).

FMOT was used to extend individual pilus as previously described [16], [54]. Briefly, a free-floating bacterium was trapped at low power by a focused laser beam and attached to a poly-L-lysine-coated large bead. Then, a small bead (3.0 µm) was captured and the trap was calibrated using the power spectrum method in order to determine the stiffness of the trap [55]. The trap stiffness was typically 130 –150 pN/µm. Thereafter, the trapped bead was moved back and forth close to the bacterium until pili attached to it. The data acquisition was started and the piezo-stage automatically set in motion in order to separate the bacterium from the small bead under velocity-clamped conditions. More or less frequently several pili were initially attached to the trapped bead. In those cases, the separation was repeated and pili detached from the bead one at a time until a single pilus remained.

In the upper urinary tract, bacteria attached to the ureter wall can be exposed to widely varying urine flows. The urine is propelled by a peristaltic activity that moves urine towards the bladder in boluses [56]. These peristaltic waves occur at a limited rate of approximately 3.3 per minute [57], with a mean bolus velocity of 2.6 cm/s. Simulations [57], [58], [59], [60] have been performed demonstrating a complex flow pattern comprising both forward and reverse flows that can expose individual bacteria attached to the wall to forces ranging throughout the entire pN to the low nN range. However, since bacteria can bind by multiple pili, and thereby an individual bacterium can redistribute a large external force among a variety of pili in such a way than none gets any excess force [40], it is conceivable that bacteria can sustain such forces. In order to facilitate the interpretation of the data, the elongation was in this work performed under steady-state conditions, *viz.* at an elongation velocity of 0.1 µm/s, which is below the so-called corner velocity, which for P pili is 400 nm/s [61], and at a constant temperature of 25°C. Since it is unlikely that the biomechanical properties of a pilus rod can be only partly compromised [17], [62] the steady-state response is considered to represent the entire biomechanical response of the pilus. This steady-state uncoiling force has previously been assessed to ∼28 pN [16], [61], [63] and it was therefore considered to be a suitable representative of the entire biomechanical properties of P pili.

### Data analysis

The binding curves obtained in SPR measurements were analyzed using the Evaluation Software Biacore X100, version 1.0. The data were analyzed using repeated measured analysis of variance (one-way ANNOVA), followed by Turkey's post hoc test using GraphPad Prism software (GraphPad Software Inc., San Diego, CA). The mean value of the force along the plateau (see figure 7A) in force-*vs.*-elongation curves obtained in FMOT were analyzed using Matlab (MathWorks, Natick, MA) and the statistical analysis (mean values of different curves, standard error of the mean (SEM) and analysis of variance) were performed using GraphPad Prism.
